# Pulmonary function decline in firefighters and non-firefighters in South Korea

**DOI:** 10.1186/2052-4374-26-9

**Published:** 2014-04-25

**Authors:** Ju-Hwan Choi, Jae-Hong Shin, Mi-Young Lee, In-Sung Chung

**Affiliations:** 1 Department of Occupational and Environmental Medicine, Dongsan Medical Center of Keimyung University, Daegu, Republic of Korea

**Keywords:** Pulmonary function test, Firefighters

## Abstract

**Objectives:**

The purpose of this study was to evaluate and compare changes to pulmonary function among firefighters and non-firefighters who were exposed to harmful substances in their work environments.

**Methods:**

Firefighters (n = 322) and non-firefighters (n = 107) in Daegu who received a pulmonary function test in 2008 and 2011 as well as a regular health examination were included. Repeated measures ANOVA was performed to evaluate the pulmonary function of the two groups over the three-year period.

**Results:**

After adjusting for age, height, body mass index, duration of exposure, physical activity, and smoking, which were statistically different between the two groups and known risk factors of pulmonary function, the forced expiratory volume in one s FEV_1_, forced vital capacity FVC, and FEV_1_/FVC% over the 3 year period were significantly lower among firefighters compared with non-firefighters.

**Conclusions:**

Evaluating the working environment of firefighters is difficult; however, our study revealed that pulmonary function declined in firefighters. Thus, more effort should be made to prevent and manage respiratory diseases early by preforming strict and consistent pulmonary function tests in firefighters.

## Introduction

Being exposed to harmful factors such as the by-products of combustion or high temperatures and being forced to work in ergonomically stressful positions are a few of the reasons why their unique work environment makes firefighters susceptible to many illnesses and disabilities such as respiratory disease, cardiovascular disease, musculoskeletal disease, and cancer [1]. According to data from the 2011 National Emergency Management Agency, the number of firefighter casualties was higher than that of 5 years ago [2]. In addition, the firefighting department had the highest casualty rate of all other departments [2]. The 2008 report from the Inspection of State Administration stated that firefighters tended to have the youngest age of death among all of the retired public servants [3]. Moreover, firefighters are exposed to many harmful substances that result in decreased pulmonary function and increased abnormal respiratory symptoms when compared with those who were not exposed to these substances [4]. For example, being exposed to a conflagration decreased overall pulmonary function among firefighters, and pulmonary function and the symptoms of this disease became more severe with increased exposure to the fire [5].

Pulmonary function decline and the development of respiratory symptoms are also significant health issues in other types of work. Being exposed to dust, gas, and fumes was correlated with respiratory disease among various kind of workers [6], In addition, a similar study found that exposure to mineral dust, silica, metal fume, iron oxide, by-products of combustion, and chemical substances decreased pulmonary function when compared with those not exposed to these harmful substances [7]. Among a group of shipyard workers, impaired pulmonary function was noted after being exposed to various metal particles such as silica glass, lead, manganese, and nickel [8]. In a cohort of workers from a refining factory, pulmonary function declined in a dose–response relationship to dust exposure each year [9].

The pulmonary function test is an important tool because it can be used to diagnose respiratory disease, evaluate its severity, observe its course, and evaluate the efficacy of any treatments. In South Korea, the pulmonary function test has been used in health examinations since 2009 [10].

With the increased understanding of the significance of respiratory disease and the impact of work environments on its development, a variety of studies have been conducted worldwide. Although studies have investigated these relationships in firefighters, few have been performed in the South Korean population. Therefore, we investigated the effect of harmful factors on pulmonary function in group of firefighters and non-firefighters.

## Materials and methods

### Study population

Firefighters (n = 322) from four fire stations in Daegu, South Korea and non-firefighters (n = 107) who participated in the 2008 and 2011 health examinations were included in our study. Those who participated in the firefighter health examinations were classified according to their affiliated department using the Fire Officers Act Amendment No. 14. Of the 322 participants, 175 (54.4%) were firefighters from the Fire Department, 80 (24.8%) were from the Administrative Department, 44 (13.7%) were from the First Aid department, and 23 (7.1%) were from the Rescue Department. Non-firefighters were categorized using the 9th Korea Standard Industrial Classification system as either working in the textile manufacturing industry (n = 23, 21.5%), plating industry (n = 17, 15.9%), printing industry (n = 22, 20.6%), general automobile repair industry (n = 17, 15.9%), or other (n = 28, 26.1%). The other group included the following industries: building and structure demolition, automobile parts manufacturing, metal casting, agriculture and forestry machinery manufacturing, ceramic manufacturing, animal food and delicatessen production, powder metallurgy manufacturing, and plastic products manufacturing.

### Variables

#### ***General and occupational characteristics***

Survey data were collected by physicians during the physical examination via a structured questionnaire, and inadequate items were modified by the interviewing physician. General characteristics collected from each participant consisted of their age, gender, smoking history/status, and frequency of physical activity. Characteristics of their occupational environment such as the name of their affiliated department, any substances they were exposed to, and the duration and frequency of each exposure were also collected. In addition, information about their past medical history and current medications were collected separately during the examination interview. Participants were classified as current smokers or non-current smokers. Data on physical activity was classified as exercising more than 30 min/day < 3 times/week or ≥3 times/week. Exposures to harmful substances were documented as the mean, minimum, and maximum value of each exposure. The number of workers exposed to each substance was also collected. The 2008 working environment measurement of Dongsan Medical Center at Keimyung University was used as a reference when calculating each participant’s exposure history.

Height and weight were measured while the subject wore light clothing and no shoes. Body mass index was calculated as weight divided by height in meters squared, and waist circumference was measured at the half point between the lowest part of the ribs and the iliac crest.

#### ***Pulmonary function test***

All pulmonary function tests were performed according to the guidelines of the American Thoracic Society and Korea Occupational Safety and Health Agency using a multi-functional spirometer HI-801 (CHEST M.I, Inc., Tokyo, Japan). Before initiating the test, the purpose and methods of the test were explained. After receiving informed consent, a nose-clip was attached to their nose while the examinees were standing. Any errors during expiration and the duration of expiration were evaluated for each subject. If the following situations occurred, the validity and reliability were checked: low peak expiratory velocity, coughing during the test, varied velocity of expiration, air leakage, inhalation during measurement, not reaching the high plateau, or a difference of less than 5% or 0.15 L between the largest forced vital capacity (FVC) value and the second largest FVC. After checking the validity and reliability of a test more than 3 times, the largest value was chosen, and these values were transformed to body temperature and pressure, saturated with water vapor. To improve the accuracy of the test, a 3-L compensator was used for correction, and the error range of the values from the correction was kept within 3.5% at 65 mL. In addition, the accuracy was kept within 0.5% of the 3-L compensator. If a group of subjects were being examined over a short time period, this correction was done more than once every 4 h, and all examiners had to have completed the pulmonary function course offered by Occupational Safety and Health Agency, which covered pneumoconiosis-specific health examination quality control. Additionally, all examiners were required to complete internal and external quality control. Forced expiratory volume in 1 s (FEV_1_) and FEV_1_/FVC% were measured and analyzed when data on FVC were collected. The Morris’s Estimation Formula was used to estimate pulmonary function and the ratio of the absolute value and estimated value were calculated [11].

### Statistical analysis

All demographic characteristics were analyzed for the firefighter group and the other worker group using independent sample t-tests and chi-squared tests, as appropriate. Independent sample t-tests were also used to analyze differences between pulmonary function for the two groups in 2008 and 2011. Repeated Measures Analysis of Variance (RMANOVA) was used to compare differences in pulmonary function measurements between the two groups over a 3 year period. Factors that were significantly different between the two groups in our analysis as well as factors that are known to affect pulmonary function in previous studies were adjusted for in the RMANOVA. The same methods were used to analyze changes in the pulmonary function between subgroups of each two group. A p-value less than 0.05 was considered statistically significant, and all analyses were done by using SPSS version 19.0 (IBM Corp., Armonk, NY, USA).

## Results

### General characteristics

Of the total 429 subjects, 322 firefighters and 107 non-firefighters, the mean age was 43.6 and 44.1 years for firefighters and non-firefighters, respectively (p = 0.645). In addition, no significant differences were found for weight change over three years between the two groups. The mean height, weight, waist circumference, and body mass index of firefighters and non-firefighters were 172.3 cm and 167.8 cm, 72.1 kg and 65.1 kg, 84.3 cm and 79.7 cm, and 24.2 kg/m^2^ and 23.0 kg/m^2^, respectively (p < 0.001). The mean duration of the exposure to harmful substances in the workplace was 6.1 years and 9.0 years for firefighters and non-firefighters, respectively (p = 0.001). There were fewer current smokers among firefighters (38/322, 11.8%) than that among non-firefighters (45/107, 42.9%) (p < 0.001). In addition, 70.5% of firefighters (227/322) exercised more than 30 min three times/week compared to 41.9% among non-firefighters (44/107) (p < 0.001) (Table 1).

**Table 1:** General characteristics of firefighters and non-firefighters

**Variables**	**Firefighters (n = 322)**		**Non-firefighters (n = 107)**		**p**
**Character**	**Mean**	SD*	**Mean**	**SD**	**p**
Age (years)	43.6	6.9	44.1	10.1	0.645
Height (cm)	172.3	4.7	167.8	6.3	< 0.001
Weight (kg)	72.1	8.0	65.1	9.1	< 0.001
Waist circumference (cm)	84.3	5.1	79.7	5.0	< 0.001
Body mass index (kg/m^2^)	24.2	2.2	23.0	2.6	< 0.001
Exposure (years)	6.1	7.8	9.0	7.6	0.001
Weight change (kg)	0.8	3.3	0.3	2.9	0.185
	n	%	n	%	
Physical activity (times/week)					
< 3	95	29.5	61	58.1	< 0.001
≥3	227	70.5	44	41.9	< 0.001
Smoking					
Yes	38	11.8	45	42.9	< 0.001
No	284	88.2	60	57.1	< 0.001

*SD standard deviation.

Harmful factors found to affect the lung function of non-firefighters include methylene diphenyl diisocyanate, toluene-2,4-diisocyanate, toluene-2,6-diisocyanate, aluminium, chromium, iron oxide, cobalt, tungsten, mineral dust, grain dust, nickel, tin, and welding fumes. According to the working environment measurement report, none of the exposures to these factors exceed the exposure limit in this study (Table 2).

**Table 2:** Reported exposure to factors harmful to pulmonary function in 2008 for the total population

**Harmful substances**	**n**	**Mean**	**Minimum**	**Maximum**	**Exposure limit**^**†**^
Methylene diphenyl diisocyanate (ppm)	37	ND^*^	ND	ND	0.005
Toluene-2,4-diisocyanate (ppm)	38	ND	ND	ND	0.005
Toluene-2,6-diisocyanate (ppm)	36	ND	ND	ND	0.005
Aluminium (mg/m^3^) (metal dust)	55	0.01156	0.00088	0.24009	10
Chromium (mg/m^3^) (metal)	29	0.00074	0.00011	0.00292	0.5
Iron oxide (mg/m^3^) (dust and fume)	35	0.03253	0.00158	0.04759	5
Cobalt (mg/m^3^) (dust and fume)	57	0.00194	0.00194	0.00194	0.02
Tungsten (mg/m^3^) (insoluble compounds)	11	ND	ND	ND	5
Mineral dust (mg/m^3^)	36	0.76358	0.239	2.014	10
Grain dust (mg/m^3^)	4	1.691	1.691	1.691	4
Nickel (mg/m^3^) (metal)	9	0.00047	0.00009	0.00169	1
Tin (mg/m^3^) (metal)	4	0.00952	ND	0.0127	2
Welding fume (mg/m^3^)	22	0.685	0.685	0.685	5

*ND Not detectable.

^†^As reported by the Ministry of Employment and Labor in 2013.

### Differences between firefighters and non-firefighters

In the 2008 dataset, no statistically significant differences were found between firefighters and non-firefighters for FEV_1_, FVC, and FEV_1_/FVC%. In the 2011 dataset, FEV_1_ was 95.48% and 98.73%, and FVC was 90.27% and 93.68% for firefighters and non-firefighters, respectively (p < 0.05) (Table 3).

**Table 3:** Pulmonary function among firefighters and non-firefighters

**Variables**	**Firefighters (n = 322)**		**Non-firefighters (n = 107)**		**p**
**Variable**	**Mean**	**SD**	**Mean**	**SD**	**p**
2008					
FEV_1_^*^ (%)	102.00	12.75	101.32	13.32	0.664
FVC^†^ (%)	95.35	10.63	96.50	11.83	0.382
FEV_1_/FVC%	82.29	5.34	81.08	6.24	0.053
2011					
FEV_1_ (%)	95.48	11.28	98.73	12.77	0.021
FVC (%)	90.27	9.28	93.68	10.26	0.003
FEV_1_/FVC%	80.52	5.64	80.35	6.47	0.791

*FEV_1_ Forced expiratory volume in 1 s.

^†^*FVC* Forced vital capacity.

### Changes to pulmonary function between the two groups in 2008 vs. 2011

**Figure 1:**
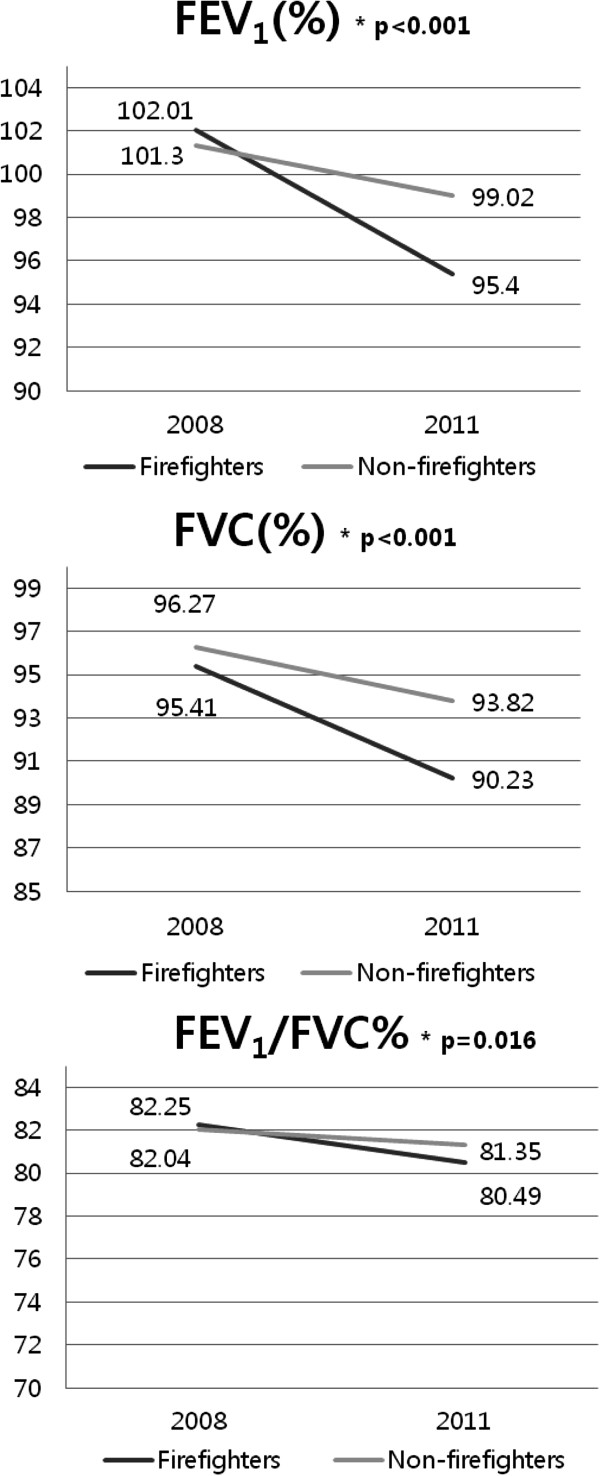
**Percentage of forced expiratory volume in one second (FEV**_**1**_**), forced vital capacity (FVC), and FEV**_**1**_**/FVC% for firefighters and non-firefighters in 2008 and 2011.** Analysis was adjusted for age, height, body mass index, years of exposure, physical activity, and smoking.

**Figure 2:**
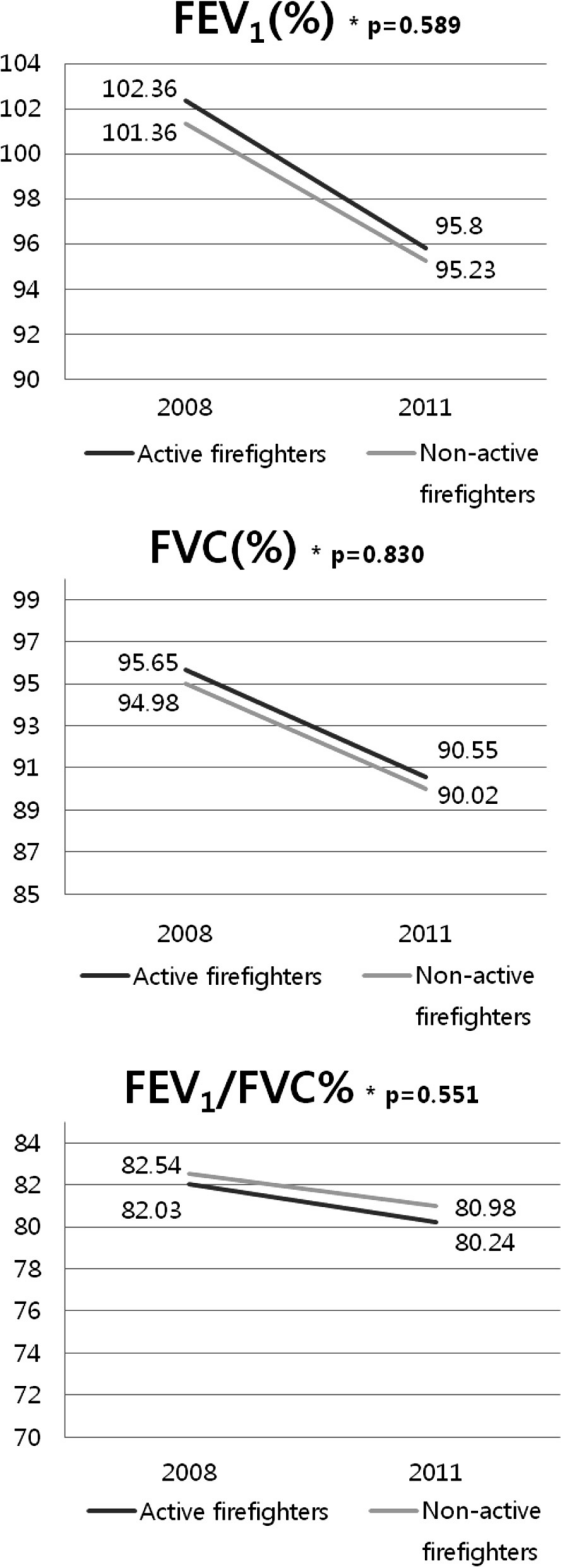
**Percentage of forced expiratory volume in one second (FEV**_**1**_**), forced vital capacity (FVC), and FEV**_**1**_**/FVC% for active firefighters and non-active firefighters in 2008 and 2011.** Analysis was adjusted for age, height, body mass index, years of exposure, physical activity, and smoking.

After adjusting for height, body mass index, exposure duration, physical activity, smoking (p < 0.05) and age in the RMANOVA, which are known to affect pulmonary function in previous studies, we found that FEV_1_, FVC, and FEV_1_/FVC% were significantly decreased in firefighters compared with non-firefighters (p < 0.05) (Figure 1). When we compared firefighters in active duty to firefighters who do not serve in active duty, no statistically significant differences were found between measurements of FEV_1_, FVC, and FEV_1_/FVC% in 2008 versus 2011 (Figure 2).

## Discussion

Inhaling smoke during fire accidents causes pulmonary damage and is known to increase morbidity and mortality in firefighters [12]. The extent of pulmonary damage differs after smoke inhalation depending on the substance and the characteristics of the gas, duration of exposure, and sensitivity of the individuals [13]. Carbon monoxide, cyanide, low oxygen concentration, isocyanate, and nitrogen dioxide cause damage to the bronchi and alveoli as well as acute respiratory dysfunction [14-16]. In addition, exposure to hydrochloric acid, nitrogen dioxide, sulfur dioxide, aldehydes, and ammonium cause inflammatory responses in the neutrophils that lead to increased respiratory sensitivity, decreased respiratory function, damage to the respiratory conduction system, chronic bronchitis, bronchiectasis, asthma, and pulmonary fibrosis [17].

In 2008, the absolute pulmonary function indices FEV_1_ and FVC among firefighters were higher than that among non-firefighters (data not shown). However, no significant differences were found for the ratio of the predicted values according to the height, age, and sex of the two groups. One reason for this finding may be that the mean age among the firefighters in our study was not significantly different from that of non-firefighters. However, firefighters tended to be taller, be exposed to harmful substances for a shorter duration, and have lower smoking rates than that of non-firefighters. A study in Australia confirms these findings as they also revealed that firefighters are taller, less likely to smoke, have better pulmonary function, and better overall health than non-firefighters tend to be [18]. In addition, healthier people tend to be chosen to become firefighters, and firefighters who maintain good pulmonary function tend to maintain employment longer than those with poor pulmonary function do [19].

In the fully adjusted RMANOVA, FEV_1_, FVC, and FEV_1_/FVC% had a significantly decreased performance among firefighters compared with non-firefighters in 2008 versus 2011. In a study on Boston firefighters, FEV_1_ and FVC decreased 68 mL and 77 mL in 1 year, respectively. In our study, firefighters were found to have a 110 mL and 103 mL decrease in FEV_1_ and FVC in 1 year, respectively. Non-firefighters had a decrease of 67 mL and 71 mL in FEV_1_ and FVC in 1 year, respectively. In both groups, a decrease of 2 times greater than the predicted value for 1 year (decrease of 30 mL) was found [20]. In our study, no significant differences were found between pulmonary function in 2008 versus 2011 between the active firefighters and non-active firefighters working in the fire departments. In South Korea, no specialized fire departments exist; therefore, workers can quickly switch roles depending upon the situation. Moreover, most workers in a firefighting department in South Korea have experience working in non-active duty (the administrative department) for several years and vice versa [4]. We also found that one person in our study population had worked in at least two other departments and served to extinguish fires. This may have influenced our finding that pulmonary function did not significantly differ among active firefighters and non-active firefighters. In addition, in the subgroup analyses of non-firefighters, pulmonary function decline within three years was not significantly different. Our study population may have not worn proper protection equipment and they would have been exposed to harmful substances for more than 9 years. As a result, pulmonary function would have declined before our data was collected. But, according to the working environment measurement report, none of the exposures to harmful factors exceed the exposure limit in this study and the exposure levels are similar, which would lead to this negative finding. However, the interpretations of our results are limited by the fact that this was a cross-sectional study. Therefore, large, prospective studies are needed, especially in firefighters, because they have an increased risk of pulmonary function decline compared to other occupations. Moreover, primary prevention and early detection for chronic diseases related to respiratory dysfunction such as asthma, reactive airway dysfunction syndrome, bronchiectasis, chronic obstructive pulmonary disease in firefighters is important; therefore, regular pulmonary function tests in firefighters have been proposed [21]. In previous studies, use of a self-contained breathing apparatus lead to a decreased incidence of pulmonary diseases [22]. In our study, firefighters did tend to wear proper protection while extinguishing a primary fire, but tended not to wear their masks in subsequent fires. Therefore, proper education may be needed.

One advantage of this study is that it compared pulmonary function in different types of workers over a three-year period, whereas previous studies tended to compare pulmonary function at one point in time. In addition, we evaluated pulmonary function decline within a given time as well as within firefighters and non-firefighters for their exposure to harmful substances. Although there are limitations to our study, studies of this kind in South Korea are difficult because anyone working within a fire department can attend to active duty, and it is difficult to analyze the diversity of dangerous substances in detail.

The first limitation of our study was that we could not measure each subject’s actual exposure, and no studies in South Korea have been able to accurately reflect exposure levels to dangerous substances among firefighters. During data collection, we noted any problems such as the number of extinguishments, which was used to estimate the level of exposure in other studies, the number of firefighters who were involved in the extinguishment field, the number outside of the field, the number that went into the field but did not actually participate in the extinguishment, etc. However, collection of this data led to difficulty in assessing the level of exposure. In addition, answers to these questions varied among the firefighters, and they had difficulty remembering the number of extinguishments they participated in at each data collection. Moreover, well-organized statistical data are lacking. Therefore, we performed our comparison study and found meaningful differences in pulmonary function between firefighters and non-firefighters, although no differences were found within the fire department. In addition, exposures to harmful substances among firefighters in our study could have been underestimated, which might mean that we were not able to reveal all significant differences. Future studies should collect data on the number of firefighters who participate in the field as well as whether protection equipment was properly worn. A previous study reported that pulmonary function decline is linked to the number of times a firefighter is exposed to a fire [20]; however, another study stated that even though protection equipment is worn, firefighters’ pulmonary function tended to declined more than that among non-firefighters group [23].

A second limitation may be time because three years may not be sufficient to assess changes in pulmonary function. However, a significant decline in pulmonary function was evident during three years and was more than that after one year among the general population.

We were also not able to analyze subjects’ exposure to individual substances. According to a previous study, carbon monoxide, hydrogen cyanide, carbon dioxide, low oxygen concentration, acrolein, formaldehyde, dioxin, dibenzofuran, isocyanate, and various microparticles were harmful to firefighters during fire accidents [24]. In another study, carbon monoxide, formaldehyde, acrolein, acetaldehyde, benzene, carbon black, graphite dust, wood dust, silica fume, and talc particles were harmful substances in fire accidents [25]. In a study of major fire accidents, firefighters who were working in the field were exposed to more xylene, lead, antimony, and perchloroethylene than the comparison group, and firefighters who were closer to the field had greater exposures to these substances than those who were farther from the field did [26]. In addition, firefighters who participated in major fire accidents had more titanium, zinc, and calcite in their sputum [27]. In South Korea, the types of harmful factors present during a fire are unknown because they can differ at each fire site [28]. Another limitation is that specimens were not collected at the individual level for non-firefighters. Therefore, we could not evaluate individual exposure levels leading to insufficient homogeneity among our collected data. We selected non-firefighters from occupational groups that were exposed to harmful substances different from the substances that firefighters were exposed to in this study. After we obtained these results, we also followed up with those in the fire department. Future studies should more accurately analyze the pulmonary function decline and exposure to harmful substances.

## Conclusion

In conclusion, firefighters had a greater degree of pulmonary function decline than that of non-firefighters. However, there are difficulties in measuring a firefighter’s working environment. Nevertheless, prevention of respiratory diseases and the early management on pulmonary function by consistent and strict pulmonary function tests on firefighters are needed. Numerous factors affect pulmonary function such as the frequency of fire exposure, wearing a respiratory protector, smoking, weight gain [29], and regular exercise [30]; therefore, pulmonary function should be maintained at a healthy level. Future studies should collect data on the harmful substances present at fire sites as well as the effects of these substances after long term exposure on pulmonary function and overall health.

## Competing interest

The authors declare that they have no competing interests.

## Authors’ contributions

JC carried out the main study. JS participated in the data collection. ML participated in the statistical analysis. IC participated in the study design. All authors read and approved the final manuscript.
